# Impairment of FOXM1 expression in mesenchymal cells from patients with myeloid neoplasms, de novo and therapy-related, may compromise their ability to support hematopoiesis

**DOI:** 10.1038/s41598-022-24644-1

**Published:** 2022-12-08

**Authors:** Giulia Falconi, Elisa Galossi, Emiliano Fabiani, Marco Pieraccioli, Serena Travaglini, Hajro Hajrullaj, Raffaella Cerretti, Raffaele Palmieri, Roberto Latagliata, Luca Maurillo, Maria Teresa Voso

**Affiliations:** 1 Department of Biomedicine and Prevention, University of Rome Tor Vergata, Via Montpellier 1, 00133 Rome, Italy; 2 Saint Camillus International, University of Health Sciences, Rome, Italy; 3 Department of Neuroscience, Section of Human Anatomy, Catholic University of the Sacred Heart, Rome, Italy; 4 Haematology Division, Ospedale Belcolle, Viterbo, Italy

**Keywords:** Gene expression analysis, Biological techniques, Molecular medicine

## Abstract

Bone marrow mesenchymal stem cells (BM-MSCs) exhibit multiple abnormalities in myelodysplastic syndromes (MDS) and acute myeloid leukemias (AML), including reduced proliferative and clonogenic capacity, altered morphology, impaired immunoregulatory properties and capacity to support hematopoiesis. Here, we investigated expression of the *FOXM1* gene, a transcription factor driving G2/M gene expression, in BM-MSCs isolated from patients with MDS and AML, de novo and therapy-related, compared to BM-MSCs isolated from healthy donors (HD). We observed a statistically significant downregulation of *FOXM1* expression in BM-MSCs isolated from MDS and AML patients, as compared to controls. In parallel, expression of FOXM1 mitotic targets (*CCNB1, CDC20, PLK1* and *NDC80*) was suppressed in patients’ BM-MSCs, as compared to HD. No differences in the expression of FOXM1 and its mitotic targets were observed in BM-mononuclear cells from the different sources. From a functional standpoint, silencing of *FOXM1* mRNA in healthy MSC induced a significant decrease in the expression of its targets. In this line, healthy MSC silenced for *FOXM1* showed an impaired ability to support hematopoiesis in vitro. These findings suggest that deregulation of *FOXM1* may be involved in the senescent phenotype observed in MSC derived from myeloid neoplasms.

## Introduction

By definition, myelodysplastic syndromes (MDS) and acute myeloid leukemia (AML) are myeloid neoplasms (MN) of hematopoietic progenitor or stem cells (HSC), in which disease initiation and progression are mostly driven by hematopoietic cell-intrinsic genetic events^1,2^. However, during the past 20 years, several observations have challenged this reductionist view, and a large number of studies have shown that de novo (*dn*) MDS and AML are associated with an abnormal bone marrow (BM) microenvironment, with mesenchymal stem cells (MSC) as a major component of this disrupted architecture^3,4^. Physiologically, BM-MSC play an essential role in normal hematopoiesis by regulating HSC proliferation and differentiation, and also provide a substantial contribution to the creation of the hematopoietic niche. The first experimental evidence supporting the crucial role of BM-MSCs in the initiation of MN derives from in vivo models, where deletion of the endoribonuclease Dicer1 restricted to the stromal compartment, was able to induce a MDS-like syndrome evolving into overt leukemia in mice^5^.

In MDS, MSCs play an important role in sustaining the disease phenotype, as demonstrated by the altered expression of adhesion proteins belonging to the PI3K/AKT and WNT/β-catenin signaling pathways^3,6–11^. From a functional point of view, BM-MSC isolated from patients with *dn* MDS and AML exhibit decreased proliferative and clonogenic capacity, altered morphology, defective osteogenic differentiation potential, impaired immune-regulatory properties, and reduced ability to support HSC growth and differentiation, as compared to normal MSC^7,–10,12^.

Moreover, several studies have reported the occurrence of non-clonal chromosomal aberrations in BM-MSCs isolated from patients with *dn* MDS and AML, which only very rarely correspond to the cytogenetic markers observed in the leukemic clone of the same individual^13,14^. However, the most common cytogenetic aberrations detected in MSCs from MDS patients are numerical, and involve chromosomes 5 and 7^15^.

In this line, Macedo et al., showed that aneuploidy increases with aging, due to a general dysfunction of the mitotic machinery in primary human dermal fibroblasts^16^. In elderly mitotic cells, increased chromosome mis-segregation correlates with an early senescence-associated phenotype, and repression of *FOXM1*, the transcription factor that drives G2/M transition. On the contrary, *FOXM1* induction in elderly fibroblasts prevents aneuploidy and ameliorates cellular aging phenotypes^16^. The function of the *FOXM1* gene has not been examined in myeloid neoplasms.

Given that MSCs from patients with MN are characterized by aneuploidy and are functionally impaired, we evaluated the role of the *FOXM1* gene in MSCs isolated from patients with de novo and therapy-related MDS and AML. To the best of our knowledge, we show for the first time that downregulation of FOXM1 and of his mitotic targets may contribute to the disrupted BM microenvironment observed in MN.

## Results

### Phenotype of MSC in myeloid neoplasms

**Figure 1:**
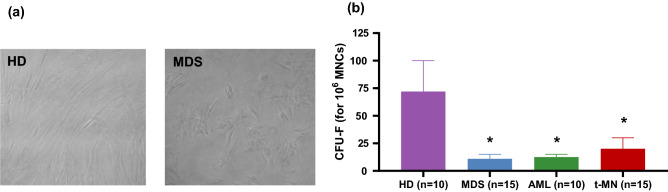
Phenotype and growth properties of patients and HD-derived MSCs. (**A**) Representative pictures of MSCs morphology in MDS patient and HD. (**B**) The Bar charts show CFU-F normalized to 1 × 106 seeded BM-MNCs (HD n = 10; AML n = 10; MDS n = 15; t-MN n = 15). Mean values are indicated by bars, while error bars indicate standard deviation. The Mann Whitney test was used to detect statistically significant differences between controls and each patient group. *p < 0.05.

We studied morphologic alterations in MSC isolated and expanded from the BM-MNC of patients with myeloid neoplasms, including AML and MDS, de novo and therapy-related. All samples were studied at the time of initial MN diagnosis. Compared to normal MSC, at the second expansion passage, patients MSC morphology was disrupted with a larger, flattened and disorganized appearance. Figure 1A shows exemplary pictures of MSC from a MDS patient and a control BM. The number of CFU-fibroblasts was also significantly reduced, with no differences between de novo and therapy-related subtypes (Fig. 1B).

### FOXM1 expression levels in AML and MDS

**Figure 2:**
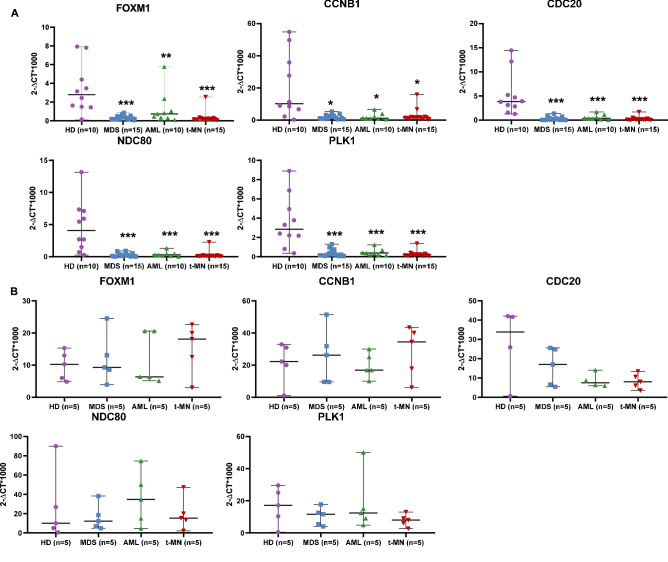
Expression levels of *FOXM1* and its mitotic genes (**A**) mRNA expression levels of *FOXM1* and its mitotic targets (*CCNB1, CDC20, NDC80, PLK1*) in MSC isolated from patients with myeloid neoplasms (MDS n = 15, AML n = 10, t-MN n = 15) as compared to healthy donors (HD, n = 10). (**B**) mRNA expression levels of *FOXM1* and its mitotic genes (*CCNB1, CDC20, NDC80, PLK1*) in MNC isolated from patients with myeloid neoplasms (MDS n = 5, AML n = 5, t-MN n = 5), as compared to healthy donors (n = 5). The gene expression values, specific for each gene, are presented as 2-ΔCt*1000, where ΔCt is Ct (test gene)—Ct (*GAPDH* housekeeping gene). The comparison between patient and control groups was performed using a nonparametric Mann–Whitney U test. *p < 0.005, **p < 0.0005.

We studied *FOXM1* mRNA expression levels in BM-MSC isolated from patients with myeloid neoplasms as compared to healthy donors. We observed a statistically significant downregulation of *FOXM1* in MSC from de novo AML and MDS, and therapy-related MN (t-MN), compared to HD (*p* = 0.0079, *p* < 0.0001, *p* = 0.0006 respectively, Fig. 2A). According to BM-blast counts, a trend for *FOXM1* downregulation was observed in MDS versus AML samples, both de novo and therapy-related (*p* = 0.0552 and *p* = 0.0593, respectively, data not shown). In AML, the lowest *FOXM1* levels were observed in therapy-related subtypes (*p* = 0.0083, Fig. 2A). On the other hand, there were no differences in *FOXM1* mRNA expression levels when comparing BM-MNCs from patients and HD, indicating that *FOXM1* downregulation is exclusive of the mesenchymal compartment (Fig. 2B).

**Figure 3:**
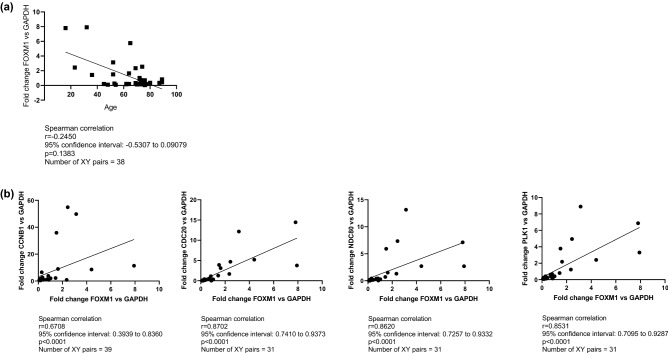
Correlation analysis of *FOXM1* expression in MSC from patients with myeloid neoplasm and healthy donors. (**A**) Patient age, (**B**)*FOXM1* target gene expression (*CCNB1, CDC20, NDC80, PLK1*). Correlation was studied using a nonparametric Spearman correlation test. The relationship between two variables is generally considered strong when their r value is higher than 0.7. Pearson r > 0 indicates a positive association.

Given the age difference between patients and controls (median age 70 years vs. 50 years, *p* < 0.0001), we assessed whether age could be a confounding factor in the analysis of *FOXM1* expression. The Spearman’s correlation test showed that *FOXM1* deregulation was not correlated with age, as showed in Fig. 3A.

**Figure 4:**
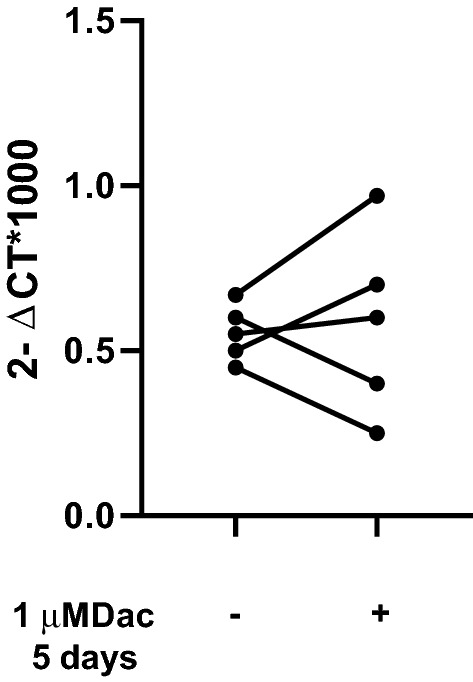
Effects of Decitabine treatment on *FOXM1* expression. FOXM1 mRNA expression levels of *FOXM1* in P2 BM-MSC isolated from 5 patients with MDS, treated with 1 µM of decitabine for 5 days. FOXM1 expression is presented as 2^−ΔCt^ × 1000, where ΔCt was Ct (*FOXM1* gene)—Ct (*GAPDH* housekeeping gene). The comparison between untreated and treated cells was performed using a nonparametric Wilcoxon matched-pairs signed rank test. *p < 0.05.

To test whether hypermethylation was the cause of FOXM1 downregulation, we treated 5 MDS-MSC samples with 1 µM decitabine (DAC), for 5 days. This treatment was unable to restore normal *FOXM1* expression levels (Fig. 4), suggesting that *FOXM1* gene regulation is not dependent on DNA methylation in the MSC context.

### Expression of FOXM1 mitotic targets

To study the functional consequences of *FOXM1* downregulation, we assessed expression levels of some of its known mitotic targets (*CCNB1*, *PLK1*, *NDC80*, and *CDC20*) (21). All genes tested were significantly downregulated in MN-MSC compared to HD-MSC, independent of the MN subtype (Fig. 2A). Levels of *FOXM1* and its mitotic targets in MSC were directly correlated (Fig. 3B), and were independent of the proportion of BM-blasts (data not shown). These quantitative analyses demonstrated that important protein players, acting from mitotic entry (*CCNB1* and *PLK1*) to anaphase onset (*CDC20* and *NDC80*), are expressed at significantly lower levels in MSC isolated from patients with MN, as compared to HD-MSC. *FOXM1* target gene levels in MNC were on the contrary similar to that of HD from the same patient group (Fig. 2B).

### FOXM1 silencing by siRNA

**Figure 5:**
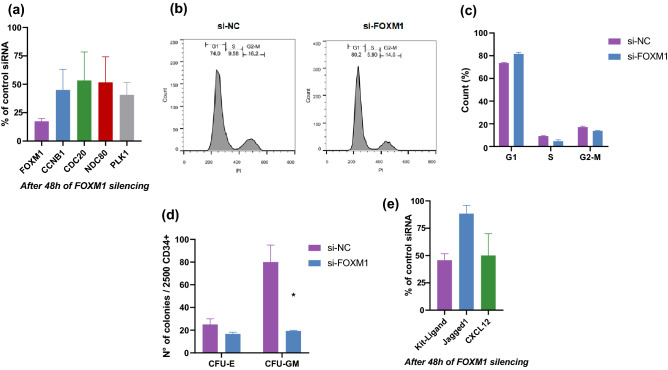
Effects of *FOXM1* silencing (**A**) Expression levels of *FOXM1* and of its mitotic targets (*CCNB1, CDC20, NDC80, PLK1*) in MSC isolated from HD (n = 5), and cultured for 48 h in the presence of 1 µM si-FOXM1^1,2,3^ RNA, as compared to treatment with control siRNA (si-NC). si-FOXM1: MSC transfected with a pool of 3 FOXM1 siRNA. (**B**–**C**) Cell cycle analysis of HD-MSC transfected with 1 µM si-FOXM1^1,2,3^ RNA and cultured for 48 h. si-NC: MSC transfected with negative control siRNA; si-FOXM1: MSC transfected with a pool of 3 FOXM1 siRNA. (**D**) In vitro colony assays. A total of 2500 CD34 + cells isolated from healthy donors (HD) were co-cultured for 10 days with HD-MSC silenced for FOXM1, and then plated in a semisolid culture system for 10–12 days. Bar plots show the number of CFU-GM and CFU-E colonies. Data are represented as mean ± SD (*p ≤ 0.05) from 3 independent experiments. CFU-GM, colony forming unit—granulocytes, macrophages; si-NC: HD-CD34 + co-cultured with MSC transfected with negative control siRNA; si-FOXM1: HD-CD34 + co-cultured with MSC transfected with a pool of 3 FOXM1 siRNA. (**E**) Expression levels of several molecules involved in HSC interaction (KitL, Jagged1, CXCL12 in MSC isolated from HD (n = 5), and cultured for 48 h in the presence of 1 µM si-FOXM1^**1,2,3**^ RNA, as compared to treatment with control siRNA (si-NC). si-FOXM1: MSC transfected with a pool of 3 FOXM1 siRNA.

To investigate the functional effects of FOXM1 downregulation, we silenced *FOXM1* mRNA, using a pool of 3 siRNA (si-*FOXM1*^*1,2,3*^) directed against different portions of the *FOXM1* gene. These tests were performed in BM-MSC from 5 HD, where expression of the *FOXM1* gene was measurable. After 48 h of si-*FOXM1*^*1,2,3*^ transfection, we observed greater than 90% decrease in the expression of *FOXM1* mRNA compared to control siRNA (Fig. 5A). *FOXM1* silencing was associated with downregulation of all target genes significantly decreased and was more pronounced for PLK1 mRNA (70%, Fig. 5A).

Considering the direct modulation of mitotic targets following *FOXM1* gene silencing, we tested whether it would interfere with the cell cycle. After 48 h of *FOXM1* silencing in HD-MSC, we did not observe differences in the distribution of cells in the different phases of cell cycle (G1, S, G2-M, Fig. 5B–C). In this line, the number of cells after 48 h of culture did not change (data not shown).

The clonogenic capacity of healthy CD34 + cells has been shown to be impaired after co-culture with MSC isolated from patients with MDS^9^. Given that *FOXM1* expression is reduced in these cells, we tested whether inhibition of *FOXM1* expression could reproduce the “MDS-MSC phenotype” in healthy MSC. To this end, we determined the frequency of myeloid progenitors using colony-forming unit assays from HD-CD34 + cells co-cultured with HD-MSC. Interestingly, CD34^+^ cells pre-incubated with *FOXM1*-deficient HD-MSC gave rise to a significantly lower number of colonies, when compared to those cultured with HD-MSC transfected with control siRNA (Fig. 5D). In detail, CFU-GM, but not CFU-E frequency was significantly reduced (median 80 vs. 19, *p* = 0.0033, vs. 25 vs. 17, *p* = 0.1). Accordingly, the expression levels of molecules involved in HSC interaction, including KIT-Ligand and CXCL2, known to be downregulated in MDS/AML-MSC^9,17^, was reduced after 48 h of *FOXM1* silencing in HD-MSC (Fig. 5E).

These data show that *FOXM1* reduction is functionally involved in the reduced ability of MDS-MSC to support hematopoiesis.

## Discussion

In the present study, we show that *FOXM1* is downregulated in BM-MSC isolated from AML and MDS patients, both de novo and therapy-related, as compared to healthy donors. *FOXM1* is a member of the forkhead transcription factor family, which plays an important role in regulating the cell cycle^18,19^. In particular, *FOXM1* controls mitotic entry through the periodic upregulation of a group of genes, that are maximally expressed during cell progression through late G2 and into M phase^20^. Two of its target genes are *CCNB1* and *PLK1*, and are part of a positive feedback loop that leads to the phosphorylation of *FOXM1,* and potentiation of its activity^21,22^. This suggests an intricate inter-regulatory relationship between *FOXM1* and *PLK1*, leading to a cell-cycle control switch. Indeed, all 4 FOXM1 mitotic targets (*CCNB1, PLK1, CDC20, NDC80*) analyzed in the present study, were significantly downregulated in BM-MSC of MN patients. SiRNA experiments showed that this deregulation depends on *FOXM1* expression, which particularly affected the *PLK1* gene, which was suppressed by 70%. The correlation between FOXM1 and PLK1 expression has also been reported by Zhang et al., in renal cancer cell lines, where PLK1 suppression induced downregulation of FOXM1 expression^23^. In turn, Dibb et al. showed that FOXM1 and PLK1 are overexpressed in patients with gastric adenocarcinomas^24^, in line with our data on myeloid neoplasms.

During recent years, novel functions for *FOXM1* have been identified in cancer cells beyond the simple acceleration of G2–M phase progression^18^. This is exemplified by FOXM1 ability to promote nuclear translocation of β-catenin in gliomas, thereby activating a WNT-regulated program^25^. In this line, we previously reported that some signaling pathways involved in multiple MSC properties, including proliferation, differentiation, and cell–cell interaction (such as PI3K/AKT and WNT/b-catenin) are deregulated in BM-MSC isolated from MDS patients^8^. In particular, we demonstrated impaired *GSK3β* expression, translating into decreased β-catenin and *WNT/β-catenin* target genes (*SOX9, EGR1, WISP1*). Since it has been recently reported that *FOXM1* may be target of WNT signaling, essential for *β-catenin/TCF4* transactivation^26^, our work converge in the same direction of a putative role of *FOXM1* as critical player in the control of WNT signaling in MN. More recently, FOXM1 was also shown to activate the Wnt–β-catenin signaling pathways in MLL-rearranged AML, by directly binding and stabilizing the β-catenin protein, thereby preserving leukemic stem cell quiescence and promoting their self-renewal^27^.

We confirm that MSC from AML and MDS cells are functionally impaired, as shown by decreased proliferative and clonogenic capacity, and altered morphology, as compared to normal MSC^7–10,12^. MSC from MN present distinct alterations in the expression of essential hematopoiesis-regulating factors such as CXCL12 and Kit-Ligand, which may underlie the deficient ability of MDS/AML-derived MSC to support healthy CD34 + HSPC^9,17^. Moreover, leukemic cells can actively reprogram healthy MSC towards a disrupted phenotype^17^. In this line, our data showed that repression of *FOXM1* by siRNA in healthy MSC is able to impair the clonogenic potential of CD34 + progenitor cells, in particular for CFU-GM, mirroring the effects observed when using MDS or AML-derived MSC. In the same line, silencing FOXM1 inhibited the proliferation and colony formation of liver cancer stem cells, and decreased expression of nuclear antigen and Ki-67 proteins^28^.

## Conclusions

Our study provides evidence that silencing FOXM1 inhibits stemness of LCSCs, by decreasing the expression of ALDH2, and represses the proliferation, migration, invasion, and tumorigenesis while inducing the apoptosis of LCSCs.

These data show that ematopoietic insufficiency in MDS is at least in part mediated via disturbed MSC functions, and opens up the possibility of targeting specific niche components to restore the correct function of MN MSCs. In this line, several authors showed that treatment of MDS-MSC may reset their normal features. In particular, Wobus et al., treated MDS-MSCs with the TGF-beta pathway inhibitor luspatercept, increasing the clonogenic potential and the migratory capacity of HSPC, both in vitro and in vivo^29^. Similarly, treatment with 5-Azacytidine improved the stemness potential and proliferation capacity of MSCs in MDS^30^, reverting the phenotype to normal in responding patients^31^. In conclusion, the present study demonstrates that impairment of *FOXM1* expression in MSC isolated from de novo and therapy-related myeloid neoplasms may underlie their defective capacity to support normal hematopoiesis.

## Methods

### Patient characteristics

The study population included 40 patients (16 females, 24 males, median age: 70 years, range: 45–89 years) with newly diagnosed myeloid malignancies (de novo AML, n = 10; de novo MDS, n = 15; and therapy-related MN, n = 15). Patients characteristics at diagnosis are illustrated in Table 1. The diagnosis was established according to standard morphologic and immunophenotypic criteria, according to the World Health Organization (WHO) classification^32^. Bone marrow cells harvested from 10 healthy hematopoietic stem cell donors (3 females and 7 males,median age: 50 years, range: 24–64 years) were used as controls. According to the declaration of Helsinki, all patients and controls gave informed consent to the study, which was approved by the Ethical Committee of Tor Vergata University.

**Table 1:** Patient characteristics.

Diagnosis	MDS (n = 15)	AML (n = 10)	t-MN (n = 15)
Age, years	74, 45–82	70.5, 54–89	73, 48–87
Sex (F/M)	6/9	3/7	7/8
Bm-blasts (%)	5, 1–10	40, 30–94	22, 5–93
**Karyotype**			
Normal	13	6	7
Del(5), del(7/7q)	1	0	1
Complex	0	1	5
Recurrent trasl	0	2	0
Other	2	1	0
Not available	0	0	2
**IPSS-R**			
Very Low/low	8		
Int-Very high	7		
**ELN 2017**			
Favourable		4	
Intermediate		5	
Unfavourable		1	

Age and BM-blasts are reported as median and range.

### Isolation and expansion of BM-MSCs

Bone marrow mononuclear cells (MNCs) were isolated by Lympholyte-H density gradient separation (Cedarlane, Euroclone, Italy). Ten millions BM-MNCs were seeded and cultured in MesenCult MSC Basal Medium (Stemcell Technologies, Italy) supplied with Mesenchymal Stem Cell Stimulatory Supplements (Stemcell Technologies) and 1% penicillin–streptomycin (Stemcell Technologies), at 37 °C and 5% CO_2_ in a humidified atmosphere. The remaining cells were stored, using Trizol reagent (Life Technologies, Italy), for subsequent RNA extraction. After 24 h of incubation, non-adherent cells were removed, and medium was changed twice a week. When adherent MSCs reached 80% of confluence, cells were detached using 0.25% trypsin–1 mmol/L EDTA (Euroclone) and further expanded for a total of five passages (P). Considering the quick exhaustion number of replications observed in culture of MSC isolated from patients with myeloid neoplasm^8^ and to limit any artifacts and clonal selection of in vitro culturing, only early cell culture passages (≤ 5) were used for all experiments. The cell morphology of each sample was examined twice a week under a phase-contrast microscope (Zeiss). BM-MSCs were characterized according to International Society for Cellular Therapy (ISCT) criteria^33,34^. The MSC phenotype of expanded cells was verified on representative samples from patients and controls by cytofluorimetry performed on trypsinized MSCs from P2 and P3. MSC immunophenotype was studied using anti-CD34, anti-CD45, anti-CD73, anti-CD90, and anti-CD105 monoclonal antibodies (MACS Miltenyi Biotec, Italy), following the manufacturer’s recommendations, and data were processed in a FACS-Canto fluorocytometer (Becton, Dickinson [BD]). Isolated BM-MSCs were positive for the MSC markers CD73, CD90, and CD105 (> 99.1% of cells), and did not express CD34 and CD45 hematopoietic antigens (< 1% of cells), according to ISCT criteria^33,34^.

BM-MSC at the second passage of expansion were incubated with MesenCult medium alone or with decitabine (1 µM) for 5 days. After 5 days of incubation, MSCs were detached using 0.25% trypsin–1 mmol/L EDTA (Euroclone) and stored, using Trizol reagent (Life Technologies, Italy) for subsequent RNA extraction.

### Colony Forming Unit- Fibroblast (CFU-F) Assay

Following the isolation by density gradient centrifugation, 10^6^ mononuclear cells were seeded and cultured in MesenCult™ MSC Basal Medium (STEMCELL Technologies), supplied with Mesenchymal Stem Cell Stimulatory Supplements (STEMCELL Technologies) and 1% penicillin–streptomycin (STEMCELL Technologies), at 37 °C and 5% CO_2_, in a humidified atmosphere. After 24 h of incubation, non-adherent cells were removed and medium was changed twice a week. After 14 days, CFU-F were washed 3 times with PBS, fixed with methanol and stained with Wright-Giemsa stain. The number of CFU-F was assessed using light microscopy (ZEISS, Germany). All experiments were performed using the biological triplicate model.

### RNA isolation, reverse transcription and Q-PCR

Total RNA was isolated from P2 MSCs and BM-MNCs of patients and controls using the RNeasy Mini Kit (Qiagen). Complementary DNAs used for reverse transcription quantitative.

(Q)-PCR were synthesized with the QuantiTect Reverse Transcription Kit (Qiagen) in accordance with the manufacturer’s instructions.

The expression levels of the mRNAs of *FOXM1* gene and its mitotic targets *CCNB1* (cyclin B1), *CDC20* (cell division cycle 20), *NDC80* (*NDC80* kinetochore complex component) and *PLK1* (polo like kinase 1) were analyzed using a semi-quantitative qRT-PCR assay (IQ™ SYBR® Green Supermix, BIO-RAD) in the QuantStudio 1 instrument (ThermoFisher), with GAPDH as the reference gene.

Real-time PCR was performed on RNA extracted from MSCs at the second passage of expansion and on BM-MNCs at the time of diagnosis. Primers used for each amplification reaction are listed in Supplementary Table 1. A melting curve (62 °C–95 °C) was generated at the end of each run to verify the specificity of the reactions. Expression of genes with a Ct > 35 cycles was considered absent. The gene expression values, specific for each gene, were expressed as 2^−ΔCt^, where ΔCt was Ct (test gene) − Ct (reference gene). A difference in gene expression between patients and controls > twofold associated with a *p* < 0.05 was considered to indicate statistical significance.

### FOXM1 specific small interfering RNAs

MSC (2 × 10^5^), isolated from 5 healthy donors, at the fifth passage of in vitro expansion, were seeded in 25 cm^2^ surface area flask at the appropriate density (60–80% of confluence). After 16–18 h, they were then transfected with silencer-GAPDH positive control siRNA (cod 4,390,849, Life Technologies, Italy), with silencer-negative control siRNA (cod 4,390,843, Life Technologies, Italy), si-*FOXM1*-1 vector (cod s5248, Life Technologies, Italy), si-*FOXM1*-2 vector (cod s5249, Life Technologies, Italy) and si-*FOXM1*-3 vector (cod s5250, Life Technologies, Italy), using Lipofectamine® RNAiMAX Transfection Reagent (Invitrogen, Waltham, Massachusetts, USA), according to the manufacturer’s protocol. A 10 μmol/L siRNA solution was prepared with deionized water. HD-MSC in the logarithmic growth stage were cultured in seven siRNA-containing medium: negative control, GAPDH positive control, *FOXM1* siRNA-1, *FOXM1* siRNA-2, *FOXM1* siRNA-3, pool of 3 *FOXM1* siRNA, and blank control (the mock-vehicle group containing only a transfection reagent without siRNA). GAPDH siRNA positive and the negative siRNA controls were used to assess the transfection efficiency.

After 24, 48 and 72 h of transfection, cells in all si-RNA groups were detached using 0.25% trypsin–1 mmol/L EDTA (Euroclone) and stored using Trizol reagent (Life Technologies, Italy), for subsequent RNA extraction.

### Cell cycle analysis

To study cell proliferation, HD-MSC cultured for 48 h in the presence of 3 *FOXM1* siRNA or the negative control siRNA were pulsed with 30 M BrdU (Sigma-Aldrich, Waltham, Massachusetts, USA) for 1 h. After three washes in PBS, cells were collected, fixed with 70% ethanol and stained with an anti-BrdU antibody (347,580, BD Biosciences) and propidium iodide (SigmaAldrich). Cell-cycle analysis was carried out using flow cytometry (FACSCalibur, BD Biosciences) according to the manufacturer’s instructions.

### Isolation and expansion of CD34 + and BM-MSC co-colture

Hematopoietic CD34 + progenitor cells, isolated from BM-MNC harvested from five healthy donors, were purified by positive selection using the midiMACS immunomagnetic separation system (Miltenyi Biotec, Bergisch Gladabach, Germany), according to the manufacturer’s instructions. The purity of CD34 + cells was assessed by flow cytometry using a monoclonal PE-conjugated anti-CD34 antibody, and resulted over 95% (range 92–98%). Purified human hematopoietic progenitor cells were grown and expanded in StemSpan™ SFEM II medium (Stemcell Technologies, Italy).

BM-MSC (7.5 × 10^4^/cm^2^) were seeded in six-well plates in MesenCult MSC Basal Medium (Stemcell Technologies, Italy). After 48 h from FOXM1 silencing in HD-MSC, BM-CD34 + cells isolated from HD were seeded directly into the MSC layer silenced using the pool of 3 *FOXM1* or negative control siRNA, at the final concentration of 1.5 × 10^4^ cells/well. Co-coltures were maintained in StemSpan™ SFEM II medium (Stemcell Technologies, Italy), for 8 days at 37 °C and 5% CO^2^. Eight days of culture were chosen for 2 reasons: most frequently described MSCs and HSCs co-culture times range from 7 to 14 days^35^, and secondly, after 8 days of co-culture there was a biological effect of MSCs on HSCs, without signs of senescence in stromal cells. With culture times longer than 14 days, MSC change their morphology and begin losing their phenotypic characteristics^35^.

### Colony forming unit (CFU) assay

The clonogenic capacity of HD-CD34^+^ cells, expanded after 8 days of co-culture with BM-MSC silenced for *FOXM1* or in the control condition, was evaluated by short-term CFU assay. Briefly, after 8 days of coculture, HD CD34^+^ cells were seeded in triplicate in a 35 mm dish (at the final concentrations of 2,5 × 10^3^ cells) and cultured in MethoCult™ H4034 Optimum (StemCell Technologies) at 37 °C, in humidified atmosphere and 5% CO_2_ for 12–14 days. After 12–14 days, the number of erythroid burst-forming unit (BFU-E), Colony Forming Unit-Erythroid (CFU-E), granulocyte–macrophage colony forming unit (CFU-GM) was determined with manual counts using an optical microscope. Biological and technical triplicate experiments were performed.

### Statistical analysis

Data are presented as median and range. Non parametric tests were used to evaluate differences among groups (Mann–Whitney U test and ANOVA one way Kruskal Wallis test).

Non parametric tests were used to evaluate differences among groups (Fisher exact test and Wilcoxon test for categorical and continuous variables, respectively). Nonparametric Spearman correlation test was used to estimate the correlation between *FOXM1* mRNA expression and patients age or *FOXM1* target genes mRNA expression. Confidence intervals were calculated at 95% level and all tests were two-sided, accepting *p* ≤ 0.05 to indicate a statistically significant difference. All statistical analyses and data graphic visualizations were performed using the GraphPad Prism Statistical PC program (GraphPad Software, San Diego, CA).

## Supplementary Information

Supplementary Information.

## Data Availability

Data will provide on request to corresponding author.
