# Supplementary material for: Impairment of FOXM1 expression in mesenchymal cells from patients with myeloid neoplasms, de novo and therapy-related, may compromise their ability to support hematopoiesis
Source: Sci Rep. 2022 Dec 8;12:21231. doi: 10.1038/s41598-022-24644-1 (PMC9732345; doi:10.1038/s41598-022-24644-1)
Supplement: Supplementary file 1 — Supplementary Information. [file 41598_2022_24644_MOESM1_ESM.pptx]

## Slide 1
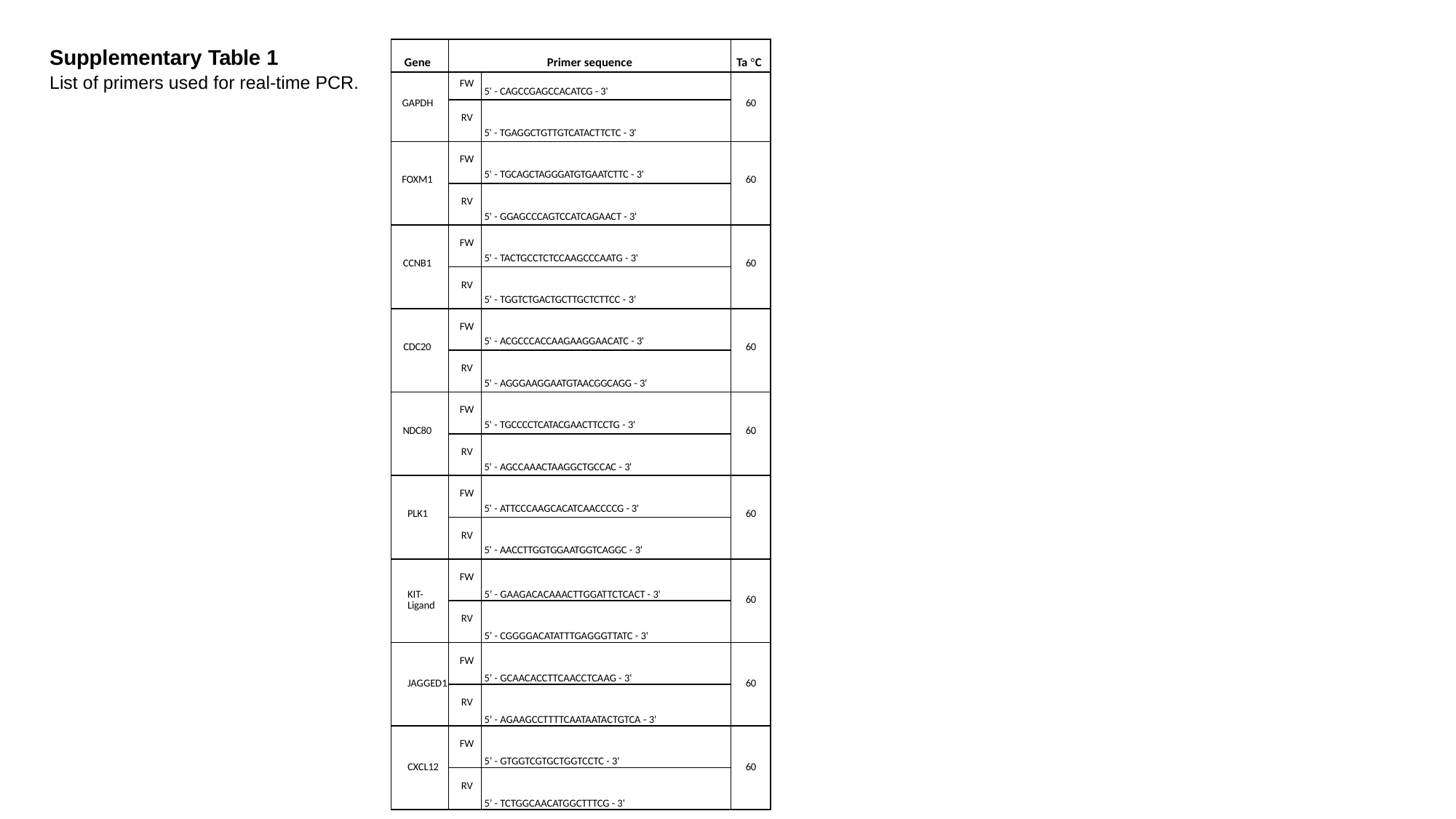

| Gene | Primer sequence | | Ta °C |
| --- | --- | --- | --- |
| GAPDH | FW | 5' - CAGCCGAGCCACATCG - 3' | 60 |
| | RV | 5' - TGAGGCTGTTGTCATACTTCTC - 3' | |
| FOXM1 | FW | 5' - TGCAGCTAGGGATGTGAATCTTC - 3' | 60 |
| | RV | 5' - GGAGCCCAGTCCATCAGAACT - 3' | |
| CCNB1 | FW | 5' - TACTGCCTCTCCAAGCCCAATG - 3' | 60 |
| | RV | 5' - TGGTCTGACTGCTTGCTCTTCC - 3' | |
| CDC20 | FW | 5' - ACGCCCACCAAGAAGGAACATC - 3' | 60 |
| | RV | 5' - AGGGAAGGAATGTAACGGCAGG - 3' | |
| NDC80 | FW | 5' - TGCCCCTCATACGAACTTCCTG - 3' | 60 |
| | RV | 5' - AGCCAAACTAAGGCTGCCAC - 3' | |
| PLK1 | FW | 5' - ATTCCCAAGCACATCAACCCCG - 3' | 60 |
| | RV | 5' - AACCTTGGTGGAATGGTCAGGC - 3' | |
| KIT-Ligand | FW | 5’ - GAAGACACAAACTTGGATTCTCACT - 3' | 60 |
| | RV | 5’ - CGGGGACATATTTGAGGGTTATC - 3' | |
| JAGGED1 | FW | 5’ - GCAACACCTTCAACCTCAAG - 3' | 60 |
| | RV | 5’ - AGAAGCCTTTTCAATAATACTGTCA - 3' | |
| CXCL12 | FW | 5’ - GTGGTCGTGCTGGTCCTC - 3' | 60 |
| | RV | 5’ - TCTGGCAACATGGCTTTCG - 3' | |
Supplementary Table 1
List of primers used for real-time PCR.
